# Characterizing and Engineering a Succinate-Responsive Biosensor System in *Escherichia coli*

**DOI:** 10.1021/acssynbio.5c00290

**Published:** 2025-08-08

**Authors:** Yusong Zou, Yuanxin Qian, Connor Parish, Logan Huddle, Yajun Yan

**Affiliations:** School of Chemical, Materials and Biomedical Engineering, College of Engineering, 1355 The University of Georgia, Athens, Georgia 30602, United States

**Keywords:** PcaR, genetic biosensors, succinate, dynamic behavior, site-directed mutagenesis

## Abstract

Metabolic engineering enables the sustainable production of valuable compounds, but challenges such as metabolic imbalances and limited regulatory tools hinder optimal yields and efficiencies. Transcription factor (TF)-based biosensors have emerged as robust solutions, allowing dynamic sensing and regulation of intracellular metabolites. However, their limited diversity often restricts their broader applications in metabolic engineering. To overcome this limitation, it is essential to develop biosensors that are responsive to central metabolic intermediates, enabling more versatile pathway control. In this study, we characterized a succinate-responsive biosensor system regulated by the IclR family TF, PcaR, and elucidated the dual-function mechanism observed in this PcaR biosensor system. Initially, we fine-tuned the expression of PcaR, fully recovering the corresponding promoter strength. Then, we discovered a dual-function mechanism of PcaR through homologue pairing, further elucidated by employing site-directed mutagenesis and promoter engineering. Meanwhile, we established a succinate-responsive biosensor library guided by PcaR–succinate complex analysis with varied dynamic ranges, identifying the superior P1-AII variant with nearly a 33-fold improvement in dynamic range. Finally, we constructed a bifunctional regulatory circuit controlled by succinate and a single regulator, demonstrating its potential for dynamic metabolic regulation. Given the primary role of succinate in central metabolism, the engineered PcaR biosensor system provides a promising tool for real-time metabolic monitoring and optimization of microbial production.

## Graphical abstract

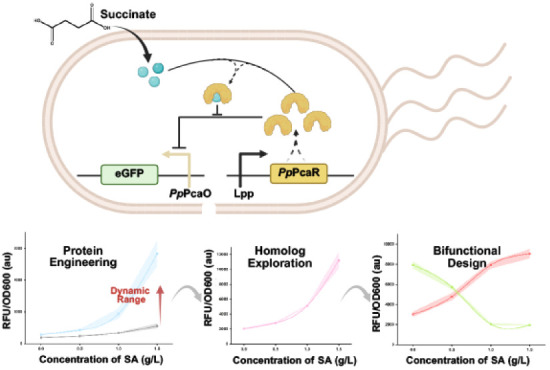

## 1 Introduction

Metabolic engineering reshapes and optimizes microbial pathways to produce valuable compounds sustainably, including biofuels, pharmaceuticals, and other industrially relevant products.^1,2^ Despite significant advancements, challenges remain in achieving optimal titer, yield, and productivity. Key obstacles include the difficulty of identifying highly efficient genes and enzymes, metabolic imbalances, and cellular burdens caused by heterologous gene expression.^3–5^ Addressing these challenges requires innovative tools that enable precise control and real-time monitoring of metabolic processes. In this case, transcription factor (TF)-based biosensors have emerged as robust solutions for dynamically sensing and regulating intracellular metabolites.^6–8^ These biosensors offer a real-time, cost-effective approach to monitoring metabolic flux, optimizing pathway performance, and enhancing microbial production capabilities. For example, biosensor-driven dynamic regulation has successfully improved the production of valuable compounds like glucaric acid,^9,10^ naringenin,^11^ muconic acid,^12^ and 4-hydroxycoumarin.^13^ However, the diversity of TF-based biosensors remains restricted, as most are designed to detect specific intermediate compounds and are therefore limited to particular metabolic pathways. This narrow diversity constrains their broader applicability in metabolic engineering, where more versatile sensing and regulatory tools are needed. To overcome this limitation, it is essential to develop biosensors responsive to metabolites integral to central metabolic pathways, which, although sometimes downstream, act as critical nodes interconnecting multiple biosynthetic routes, thus enabling more versatile and practical pathway control.

By targeting key metabolites in central metabolism, these biosensors can enable broader control over cellular metabolism, enhancing pathway optimization and bioproduction efficiency.^14–16^ Recent studies have demonstrated the potential of central metabolism-responsive biosensors in dynamically regulating metabolic fluxes. For example, a lactate-responsive biosensor based on LldR has been developed to regulate lactate metabolism in *Escherichia coli* (*E. coli*), improving bioconversion efficiency.^17^ Similarly, a biosensor responsive to pyruvate, engineered using the PdhR regulator, has been employed for adaptive laboratory evolution to enhance glycolytic flux.^10,18^ A malonyl-CoA biosensor derived from FapR has also facilitated the production of polyketide-derived chemicals.^19^ Despite these advances, the development of central metabolism-responsive biosensors is still in its early stages, with current efforts primarily focused on a limited number of metabolites. The primary challenge lies in the scarcity of transcriptional regulators naturally capable of sensing central metabolites, necessitating further exploration and engineering of novel regulatory elements. Previous studies have reported an IclR family transcription factor, PcaR, which is derived initially from *Pseudomonas putida* KT2440 (locus tag: PP_3015) (*P. putida*) and can sense the central metabolite succinate.^20^ Nevertheless, the PcaR transcription factor has not been systematically explored for its binding mechanism and engineering potential in synthetic biology. Although prior studies have evaluated its ligand responsiveness^25^ and demonstrated initial engineering efforts to alter its effector specificity, such as enhancing adipic acid recognition,^21^ an integrated analysis combining promoter control, mutational tuning, and regulatory design remains underdeveloped.

This study focused on characterizing a succinate-responsive TF-based biosensor system regulated by PcaR in *E. coli*. Initially, the wild-type *Pp*PcaR biosensor system showed limited output strength. Upon fine-tuning the expression of the regulator, the strength of *Pp*PcaO could be fully recovered with the induction of succinate. Then, we identified the potential dual function of PcaR after pairing it with a PcaR homologue, *Ps*PcaR. The dual-function mechanism was further investigated and elucidated through site-directed mutagenesis and promoter engineering. Meanwhile, we established a succinate-responsive biosensor library with various dynamic ranges for broader engineering potentials. Specifically, the variant P1-AII exhibited the most expanded dynamic range (from 2057 to 11202 au), which is a 32.9-fold improvement over the wild-type PcaR biosensor system. Ultimately, we constructed and characterized a bifunctional circuit controlled by a single regulator and succinate, demonstrating the potential of an engineered PcaR biosensor system in dynamic regulation cases. Given succinate’s central role in the TCA cycle, the engineered PcaR biosensor system offers a promising tool for real-time succinate detection, dynamic metabolic regulation, and enhanced microbial production of succinate-derived compounds.

## 2 Method and Material

### 2.1 Bacterial Strains and Chemicals

The bacterial strains used in this study are listed in Table S1. The research used Luria–Bertani (LB) medium as the growth medium for *E. coli* and selected ampicillin and kanamycin as antibiotics based on the resistance genes. The concentration of antibiotics in the medium was at final concentrations of 100 and 50 μg/mL. The plasmids were constructed using *Escherichia coli* strain XL1-Blue and tested using BW25113 F’ as the host. 100 mg of adipic acid, citrate, glutaric acid, maleic acid, malonic acid, and succinate were dissolved in 1 mL of methanol to prepare inducers with a concentration of 100 g/L. Succinate and other carboxylic acids were purchased from Sigma-Aldrich company.

### 2.2 DNA Manipulation

All manipulations of DNA were conducted using standard molecular cloning protocols.^22^ Plasmids used in this work are listed in Table S1. This research used plasmids pHA (high-copy plasmid)^11^ and pMK (medium-copy plasmid)^13^ as the framework for constructing biosensor systems. These plasmids also carry a synthetic multicloning site (MCS) that sequentially contains the recognition sites of Acc65I, NdeI, BsrGI, *Sal*I, ClaI, *Hin*dIII, NheI, *Bam*HI, and MluI.^23^ In this study, the *Pp*PcaR gene (locus tag: PP_3015) from *Pseudomonas putida* KT2440 was amplified and cloned into the plasmid system for biosensor construction. For the characterization of the PcaR biosensor system, the promoter PcaO homologues were inserted into plasmid pHA-MCS separately by XhoI and *Sal*I, resulting in plasmids pHA-*Pp*PcaO-eGFP and pHA-*Ps*PcaO-eGFP. To express the regulator protein, a series of lpp promoters harboring PcaR homologues were amplified from their genomes and inserted into plasmids pMK-MCS using XhoI and *Sal*I, respectively (seeTable S2 for a list of all PcaR homologue sequences used in this study). Site-directed mutagenesis of PcaR was conducted by overlap extension PCR using plasmids pMK-lpp0.2-*Pp*PcaR and pMK-lpp0.2-*Ps*PcaR as the templates, respectively. To construct hybrid promoters, pHA-pL_lacO1_-eGFP was replaced with the pL_lacO1_ promoter sequence to generate the parental plasmid pHA-pL-eGFP.^24^ Three sites on the pL promoter were selected to substitute the PcaO binding boxes (∼15 bp): before the −35 region (Position 1), between the −35 and −10 regions (Position 2), and behind the −10 region (Position 3). All of the hybrid promoters were amplified from their corresponding promoter sequences and inserted into the plasmids pHA-MCS-eGFP using XhoI and *Eco*RI, respectively, resulting in pHA-*Pp*HP1-eGFP, pHA-*Pp*HP2-eGFP, pHA-*Pp*HP3-eGFP, pHA-*Ps*HP1-eGFP, pHA-*Ps*HP2-eGFP, and pHA-*Ps*HP3-eGFP, respectively. For the characterization of the self-regulated bifunctional networks, a red fluorescent protein-encoding gene (RFP) was amplified and assembled into two engineered biosensor systems using SacI and SpeI, yielding pHA-*Ps*HP3-eGFP-*Ps*HP1-RFP and pHA-*Ps*HP3-eGFP-*Pp*HP3-RFP, respectively.

### 2.3 Cultivation Conditions

Three randomly picked single transformants were inoculated into test tubes containing 3.5 mL of LB medium and appropriate antibiotics. All tested strains were cultured in the New Brunswick Excella E24 shaker (shaker diameter 19.1 mm) at a constant temperature of 37 °C and a shaking speed of 270 rpm. After overnight incubation, a 150 μL portion of seed culture was transferred into fresh LB medium containing the same antibiotics and incubated at 37 °C in the shaker. After 1.5 h, when the OD_600_ reached around 0.4, varying concentrations of substrates (succinate or other dicarboxylic acids) were added. After an appropriate cultivation time, the cell density (OD_600_) and fluorescence intensity of the strains.

### 2.4 Fluorescence Assays

The samples were diluted five times with water (40 μL sample with 160 μL water) and transferred into a black 96-well plate with a clear bottom (Corning 3603) for fluorescence analysis using Biotek’s Synergy HT flatbed reader. Excitation filters of 485/20 nm and emission filters of 528/20 nm were used to detect eGFP and RFP fluorescence intensity, and cell density (OD_600_) was measured simultaneously.

### 2.5 Modeling Analysis

The analysis was based on the crystal structure of the *Pp*PcaR–succinate complex (PDB ID: 8eju). Protein sequence alignment between *Pp*PcaR and *Ps*PcaR was conducted using the Expasy SIM alignment tool (https://web.expasy.org/sim/). Residues in *Ps*PcaR that aligned with known succinate-binding residues in *Pp*PcaR were inferred as putative binding sites and selected for site-directed mutagenesis. Structural positions were interpreted with reference to PyMOL visualization of the 8eju structure.

### 2.6 Statistics

The sample size was not predetermined by using any statistical method. For eGFP and RFP regulation assays and shake flask experiments, all data were reported as the mean ± standard deviation of biological triplicates (*n* = 3) and were presented in the corresponding figure legends. Data analysis was performed by using Microsoft Excel. The colonies used for data collection were randomly selected from the agar plates. The investigators were not blinded to allocation during experiments or outcome assessment.

## 3 Results

### 3.1 Establishment and Characterization of the PcaR Biosensor System

The transcription factor (TF), PcaR, belongs to the IclR family, whose members regulate diverse cellular processes such as metabolism and quorum sensing.^25^ In *P. putida*, PcaR binds to various promoters and is essential for the degradation of *p*-hydroxybenzoate.^24^ Within the *pca* operon, *pcaBDC*, *pcaF*, and *pcaO* (or *pcaIJ*) are transcriptionally induced by β-ketoadipate in association with the regulator PcaR.^20,26,27^ Previous *in vitro* studies revealed that PcaR binds its operator binding site (GTTCGATAATCGCAC) within the PcaO promoter, inhibiting transcription.^28^ In the presence of succinate, PcaR undergoes oligomerization beyond its homodimeric form, which not only lifts transcriptional repression but can also promote activation depending on the promoter context, likely by enhancing RNA polymerase engagement.^24,28,29^ To investigate the dynamic properties of the PcaR biosensor system in *E. coli*, we constructed genetic circuits incorporating key regulatory elements in the BW25113 F’ strain by using a double-plasmid configuration (Figure 1a). Specifically, we inserted PcaR downstream of a constitutive promoter, lpp1.0, in the medium-copy plasmid pMK-MCS. The corresponding promoter PcaO was used to control the expression of an eGFP reporter gene in the high-copy plasmid pHA-MCS. As the positive control (PC), the fluorescence intensity of the strain harboring pHA-PcaO-eGFP was measured, yielding a normalized intensity of 5270 ± 297 au (Figure 1b). Upon coexpression with pMK-lpp1.0-PcaR, the fluorescence intensity decreased to 384 ± 19 au (Figure 1b), indicating that PcaR can inhibit PcaO transcription. Importantly, succinate, when supplied exogenously, enters *E. coli* cells primarily through specific C4-dicarboxylate transporters, particularly the Dcu family proteins (e.g., DcuA, DcuB, and DcuC), thus enabling intracellular availability of succinate and subsequent biosensor activation.^30^ Biosensor induction was assessed at succinate concentrations of 0.5, 1, and 1.5 g/L, with 1.5 g/L set as the upper limit due to succinate toxicity (Figure S1). Specifically, upon the addition of 0–1.5 g/L succinate, the dynamic range of the lpp1.0-PcaR group ranged from 384 to 662 au (Figure 1c). We also tested sodium succinate, a commonly used ionic salt form that dissociates to release bioavailable succinate anions, to evaluate whether it could serve as a practical substitute for succinate and expand the biosensor’s responsive scope. However, the system did not show a measurable response under the tested conditions (Figure S2), which may be due to differences in uptake efficiency or transporter compatibility in *E. coli*.

**Figure 1:**
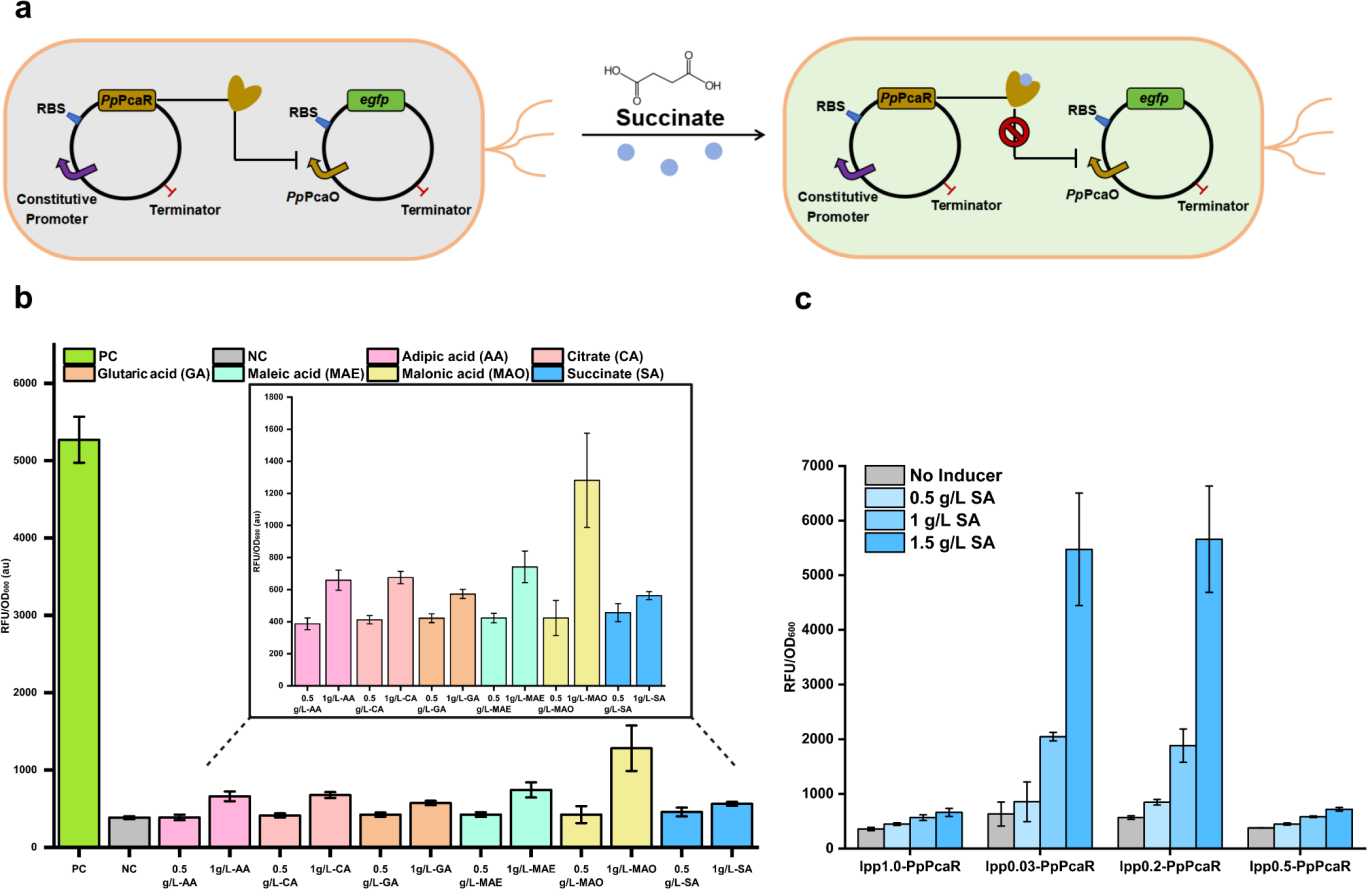
*Pp*PcaR regulation system development and characterization. (a) Establishment of *Pp*PcaR regulation system in *E. coli*. The transcription factor gene (*pcaR*) was placed under the control of a constitutive *lpp* promoter (e.g., *lpp*1.0, *lpp*0.5, *lpp*0.2, or *lpp*0.03), while the reporter gene (*egfp*) was driven by the PcaO-regulated promoter. Different *lpp* promoter strengths were used to modulate PcaR expression and assess their impact on biosensor performance (see panel c) (b) Investigation of the substrate scope of the *Pp*PcaR biosensor system. (c) Fine-tuning of *Pp*PcaR-*Pp*PcaO biosensor system by lpp promoter variants. All tests were performed with three independent biological repeats, and error bars indicate standard deviations (SD).

Beyond its primary inducer, succinate, PcaR is also considered a universal dicarboxylic acid-responsive regulator. To explore its responsiveness, we tested four additional dicarboxylic acids and one tricarboxylic acid (Figure 1b). These carboxylic acids are essential intermediates in central metabolism or precursors for value-added compounds. Compared to the uninduced control, all tested substrates enabled an increase in the eGFP expression level upon induction. Significantly, the best effector was determined to be malonic acid (MAO), which resulted in a 3.34-fold increase in the eGFP expression level upon induction with a concentration of 1g/L, followed by maleic acid (MAE) (1.93-fold), adipic acid (AA) (1.72-fold), citrate (CA) (1.65-fold), glutaric acid (GA) (1.49-fold), and succinate (1.47-fold), respectively (Figure 1b). However, the toxicity of malonic acid impaired cell performance, leading to its highest dynamic fold change. These findings suggest potential cross-talk effects if this biosensor system is applied in dynamic regulation. So far, the limited dynamic fold change induced by succinate remains a concern for the PcaR biosensor system.

We speculated that excessive PcaR expression contributes to the limited dynamic fold change. To examine this, we adopted a previously constructed lpp promoter library containing variants with different strengths to fine-tune the expression level of PcaR.^31^ To weaken PcaR-PcaO interactions, we selected lpp variants with reduced strengths, including lpp0.5, lpp0.2, and lpp0.03. The highest activation strength of the PcaO promoter with lpp0.2- and lpp0.03-controlled PcaR was 5659 and 5473 au, respectively, after adding 1.5 g/L succinate (Figure 1c). Both groups achieved full PcaO induction, highlighting the benefits of fine-tuning PcaR expression. However, the lpp0.03-controlled PcaR group exhibited a higher basal level than the lpp0.2 group (Figure 1c). Meanwhile, the dose–response of the lpp0.5-PcaR group was displayed in a manner similar to that of the lpp1.0 group (Figure 1c). These results demonstrate that using weaker promoters, such as lpp0.03 and lpp0.2, exhibited a greater fold change compared to *lpp*1.0, suggesting that reducing PcaR abundance alleviates potential promoter repression and allows more effective activation upon ligand binding. These results highlight the importance of transcription factor tuning as a strategy to enhance biosensor responsiveness and demonstrate that promoter engineering can improve performance, even when using the native PcaO regulatory element. However, insufficient PcaR availability would increase basal expression as well.

### 3.2 Exploration of the PcaR Biosensor System by Protein BLAST

Given the advancements in the characterization of the PcaR biosensor system derived from *P. pudita*, we plan to explore more potential functional homologues from other microbes. Thus, we conducted protein BLAST analysis for PcaR derived from *P. pudita* (*Pp*PcaR). We identified an 86% identical homologue (NCBI reference ID: AZO91630.1) in *Pseudomonas stuzeri* (hereafter named *Ps*PcaR). Next, we sought to identify the corresponding promoter and operator binding site of *Ps*PcaR. The *Ps*PcaR-corresponding promoter sequence, *Ps*PcaO, was identified within the *Ps*Pca operon, located from the −69 to +4 region (see Table S2 for a list of PcaR homologue sequences used in this study). Using the operator binding site of *Pp*PcaR as the template, the operator binding site of *Ps*PcaR (GCCTCGATAAGCGAG) was determined within *Ps*PcaO. Due to the high sequence similarity, we hypothesized that *Ps*PcaR possesses a similar function to *Pp*PcaR. To test this, we constructed the *Ps*PcaR biosensor system in the BW 25113 F’ strain according to the optimized *Pp*PcaR biosensor configuration (Figure 2a). We first investigated the original dose–response of the *Ps*PcaR biosensor system. The fluorescence intensity of *Ps*PcaO could reach 315 ± 8 au without the expression of *Ps*PcaR, which is lower than that of *Pp*PcaO (Figure S3). Surprisingly, when we overexpressed the regulator *Ps*PcaR, the fluorescence intensity gradually increased with the addition of succinate (Figure 2b). This result demonstrated that the functionality of *Ps*PcaR served as the activator in the *Ps*PcaR-*Ps*PcaO biosensor system, which is different from the functionality of *Pp*PcaR. To further examine the functionality of the *Ps*PcaR-*Ps*PcaO biosensor system, we also investigated its dose–response under the control of lpp1.0. Although the promoter intensity was slightly repressed without adding succinate, the highest fluorescence intensity was higher than the original *Ps*PcaO strength, indicating the activation function of *Ps*PcaR (Figure S4). These data further confirmed the different mechanisms between *Pp*PcaR and *Ps*PcaR biosensor systems.

**Figure 2:**
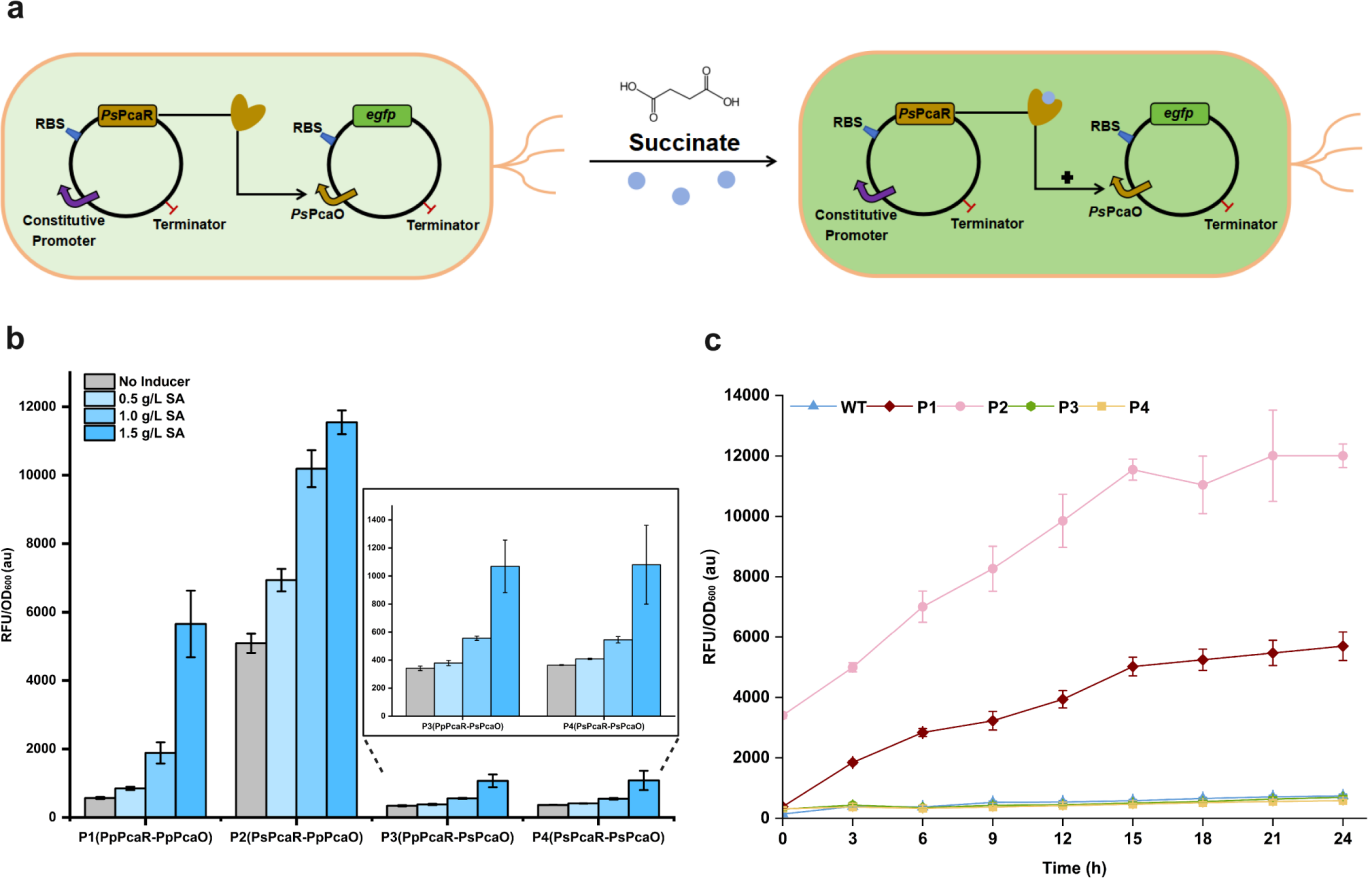
*Ps*PcaR regulation system development and characterization. (a) Establishment of *Ps*PcaR regulation system in *E. coli*. (b) Dose–response analysis of paired PcaR biosensor systems. The transcription factor genes were expressed under the control of the *lpp0.2* promoter.(c) Time-course experiment of paired PcaR biosensor systems under the induction of 1.5 g/L succinate. All tests were performed with three independent biological repeats, and error bars indicate standard deviations (SD).

To investigate the reason for such different regulator functionality, we paired PcaR homologues and their corresponding promoter sequences, obtaining four PcaR biosensor groups (P1: *Pp*PcaR-*Pp*PcaO; P2: *Ps*PcaR-*Pp*PcaO; P3: *Pp*PcaR-*Ps*PcaO; P4: *Ps*PcaR*-Ps*PcaO). Notably, *Pp*PcaR did not show any repression when paired with *Ps*PcaO, and the strength of *Ps*PcaO still increased in P3 (ranging from 341 to 1069 au) (Figure 2b). In addition, *Ps*PcaR exhibited consistent functionality pairing with *Ps*PcaO or *Pp*PcaO. Specifically, the dynamic range of P2 spanned from 5,629 to 11,547 au, representing a 2.2-fold improvement over the original *Pp*PcaO strength (Figure 2b). To further examine the dynamic behavior of these systems, we conducted a time-course experiment comparing all four PcaR–promoter pairings alongside the original lpp1.0-controlled *Pp*PcaR biosensor configuration. As the data show in Figures 2c andS5, with the induction of succinate, the strength of *Pp*PcaO could be fully recovered by fine-tuning the expression of *Pp*PcaR, and it could also be further enhanced when paired with *Ps*PcaR. This result indicated that *Pp*PcaR possesses dual functionality: repression and activation. *Pp*PcaR functioned as a promoter repressor when paired with *Pp*PcaO and as a promoter activator when paired with *Ps*PcaO. This context-dependent switching behavior suggests that promoter architecture—particularly the positioning of the binding site relative to core promoter elements—may influence whether *Pp*PcaR recruits or inhibits RNA polymerase. Moreover, *Ps*PcaR only served as a promoter activator. Besides PcaR, some other IclR TFs function as activators, repressors, or dual-function regulators.^32^ Generally, IclR family repressors bind to the target DNA to occlude RNA polymerase (RNAP) complex binding or inhibit the progression of the RNAP complex through the open complex.^31^ In contrast, activators are involved in the recruitment of RNAP or access of the RNAP to the DNA for transcription.^33^ However, the specific dual-function mechanisms remain unclear, since the preferred DNA locations and ligand pocket for binding remain unknown. Thus, much remains to be elucidated with respect to the PcaR–succinate and PcaR–DNA basic mechanisms.

### 3.3 Investigation of PcaR–Succinate Complex and Site-Directed Mutagenesis

First, we investigated the interaction between PcaR and the ligand, aiming to find the critical binding residues causing the dual function. Here, we selected *Pp*PcaR as the template. *Pp*PcaR has an *N*-terminal helix-turn-helix (HTH) DNA-binding domain (DBD), which dimerizes or tetramerizes to bind to target DNA, and a C-terminal ligand-binding domain (LBD) connected to the HTH domain by an α-helix and loop (Figure 3a). To investigate the functional region of *Pp*PcaR, the molecular docking model of the *Pp*PcaR–succinate complex (PDB ID: 8eju) was used as the standard.^24^ Assisted by PyMOL analysis, five binding residues (Y151, T177, S240, N257, and S259) were screened out, which formed hydrogen bonds with succinate (Figure 3a). In terms of the results of the *Pp*PcaR–succinate binding motif and alignment, five binding residues (A122, S148, A207, I223, and I225) were selected as potential critical binding residues within the *Ps*PcaR–succinate complex (Figure S6). We observed significant differences in the critical binding residues between the *Pp*PcaR–succinate and *Ps*PcaR–succinate complexes, which may contribute to their distinct regulatory functions. To test this hypothesis, we performed site-directed mutagenesis on key residues within the *Pp*PcaR–succinate complex in an attempt to alter its function from a repressor (*Pp*PcaR) to an activator (*Ps*PcaR). Based on the alignment results, we inserted the mutations Y151A, T177S, S240A, N257I, and S259I into the engineered *Pp*PcaR biosensor system (P1 group), respectively. P1-T177S and P1-S240A exhibited similar dose–response profiles to the wild-type P1. Notably, in the P1-Y151A group, the highest output strength (7562 au) increased over the original promoter intensity (5270 au) (Figure 3b). Meanwhile, the basal expression of the P1-Y151A group also improved to 1976 au, indicating that *Pp*PcaR (Y151A) possesses a dual function (Figure 3b). On one hand, transcription of *Pp*PcaO could be repressed when the biosensor system was exposed to a low concentration (<1 g/L) of succinate. On the other hand, the transcription of *Pp*PcaO could be enhanced when the biosensor system was exposed to a high concentration (>1 g/L) of succinate. In addition, in the P1-II (N257I/S259I) group, the basal expression (4939 au) showed a similar level to the original promoter intensity, and the highest output strength (10396 au) exhibited a 1.97-fold improvement (Figure 3b). Given the lower basal level of Y151A, we constructed a three-site mutant P1-AII (Y151A/N257I/S259I) to further expand the dynamic range. As expected, the basal expression of the P1-YNS group decreased compared to the double-site mutant, while the output strength remained at a comparable level (Figure 3b). The dynamic range of the P1-AII group responded from 2057 to 11202 au, representing a 32.9-fold improvement over the wild-type PcaR biosensor system. We also constructed several additional mutants to cover all possibilities; however, no mutant showed better performance than the three-site mutant (Y151A/N257I/S259I) (Figure S7). What’s more, we also inserted the mutations A122Y, S148T, A207S, I223N, and I225S into the engineered *Ps*PcaR biosensor system (P4 group), respectively, attempting to alter its function from an activator (*Ps*PcaR) to a repressor (*Pp*PcaR). As the data shown in Figure S8, the dose–response of all P4 mutants did not show any observable difference, indicating that the interaction between *Ps*PcaR and succinate did not play a primary role in changing regulator functionality. Nevertheless, Y151, N257, and S259 exhibited a major influence in the *Pp*PcaR–succinate complex, resulting in an enhanced dose–response.

**Figure 3:**
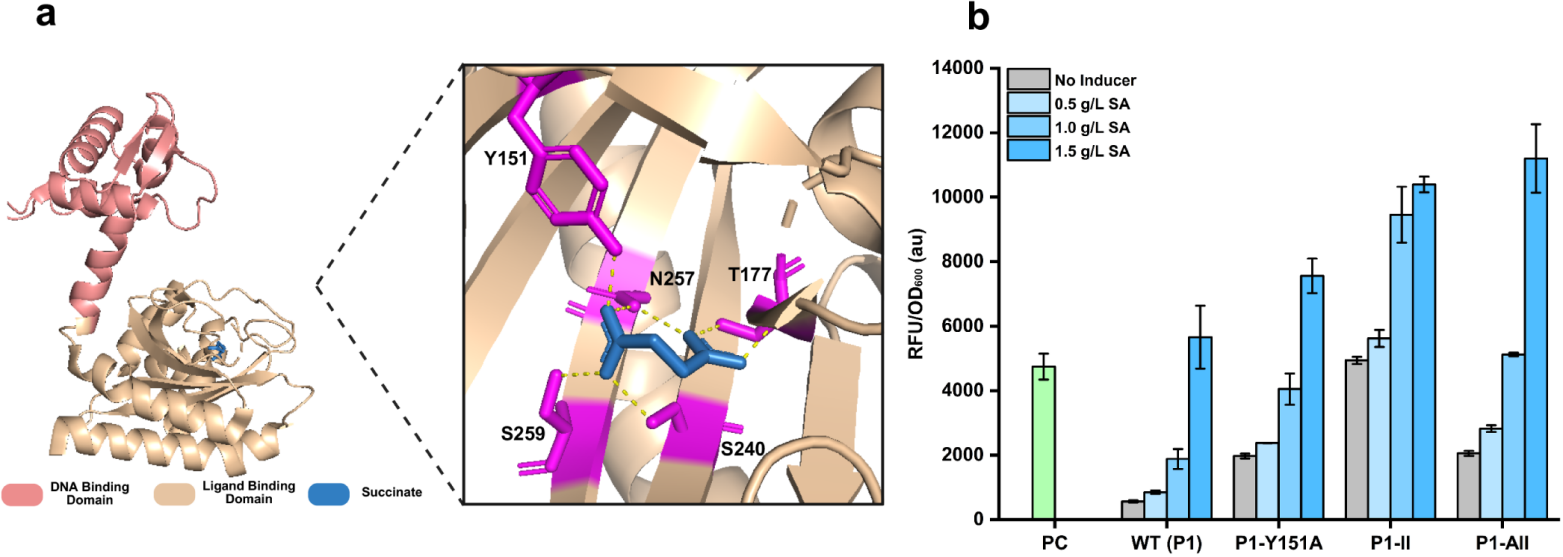
Investigation of PcaR regulation mechanism. (a) Schematic of the PcaR structure and zoomed-in views of its potential ligand-binding residues (PDB ID: 8eju). (b) Dose–response of *Pp*PcaR variants upon site-directed mutagenesis. PC, positive control, intensity of *Pp*PcaO. WT (P1), *Pp*PcaR-*Pp*PcaO. P1–Y151A, *Pp*PcaR (Y151A)-*Pp*PcaO. P1–II, *Pp*PcaR (N257I/S259I)-*Pp*PcaO. P1-AII, *Pp*PcaR (Y151A/N257I/S259I)-*Pp*PcaO. All tests were performed with three independent biological repeats, and error bars indicate standard deviations (SD).

### 3.4 Investigation of the PcaR–DNA Complex by Constructing Hybrid Promoters

After exploring the LBD of PcaR, we further investigated the DBD of this PcaR biosensor system. The operator binding boxes of both *Pp*PcaR and *Ps*PcaR have been characterized, confirming their function in binding to the target DNA sequence. Based on the noneffect of site-directed mutagenesis of *Ps*PcaR, we speculated that the interaction between the regulator and the binding box would also influence the differing functional performance of PcaR. To test this, we placed the binding boxes of *Pp*PcaR and *Ps*PcaR into a constitutive promoter at different locations, respectively (Figure 4a). Here, we chose the strong constitutive promoter PL from the phage lambda as a proof-of-concept demonstration to construct hybrid promoters. We hypothesized that placing binding boxes at different locations could shift the PcaR function.

**Figure 4:**
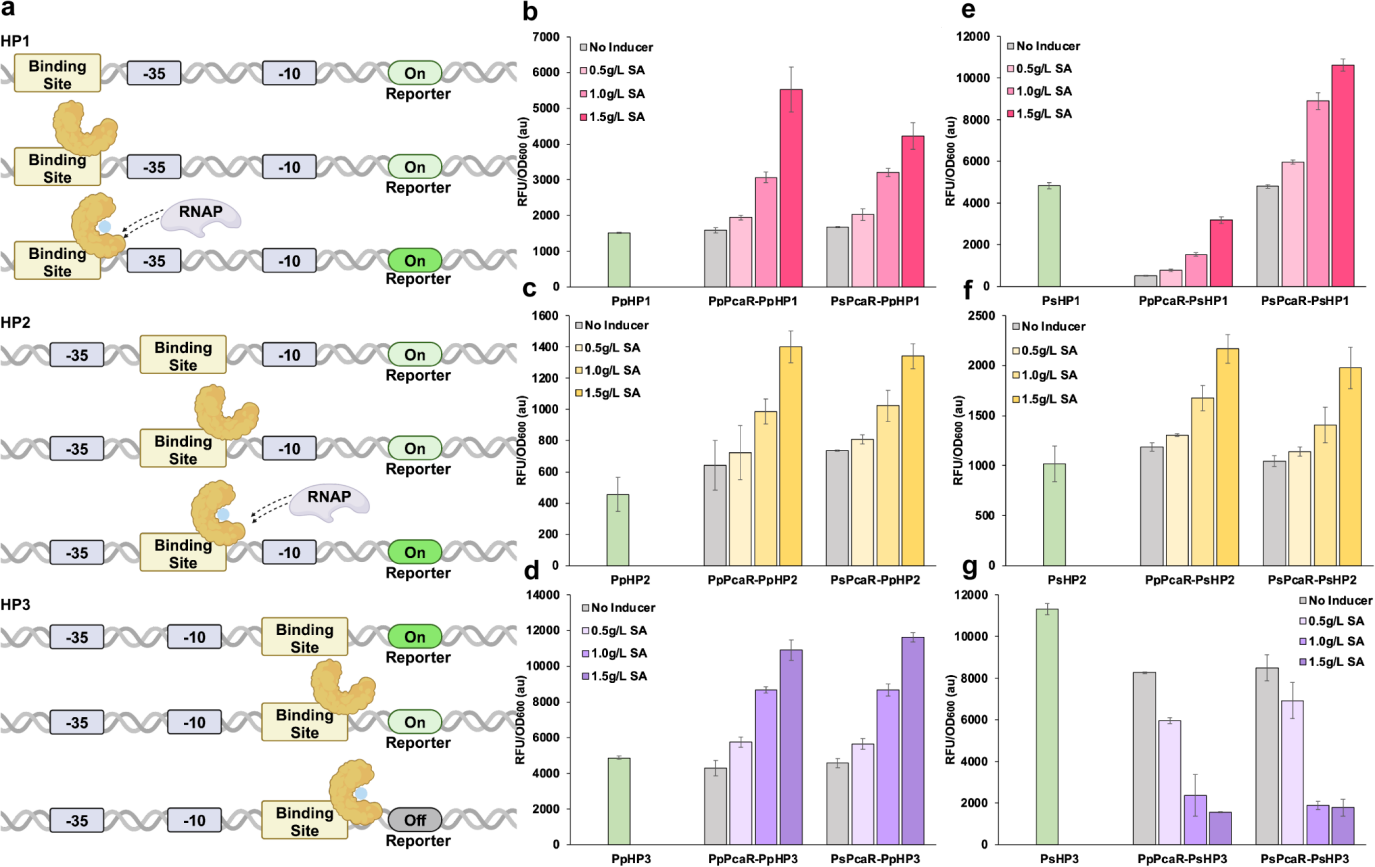
Investigation of PcaR–DNA binding mechanism using hybrid promoters. (a) Schematic illustration of hybrid promoter design based on the λ phage PL core promoter. Binding boxes from *Pp*PcaO or *Ps*PcaO were inserted at three positions: upstream of the −35 region (Position 1), between the −35 and −10 regions (Position 2), and downstream of the −10 region (Position 3). RNAP, RNA polymerase. (b–d) Dose–response curves for hybrid promoters containing the *Pp*PcaO binding box at Positions 1 (*Pp*HP1), 2 (*Pp*HP2), and 3 (*Pp*HP3), respectively, in combination with either *Pp*PcaR or *Ps*PcaR. (e–g) Dose–response curves for hybrid promoters containing the *Ps*PcaO binding box at Positions 1 (*Ps*HP1), 2 (*Ps*HP2), and 3 (*Ps*HP3), respectively, in combination with either *Pp*PcaR or *Ps*PcaR. All data are presented as RFU/OD_600_ to normalize for cell density. Succinate was added at increasing concentrations (0, 0.5, 1.0, and 1.5 g/L) to evaluate biosensor induction. All tests were performed with three independent biological repeats, and error bars indicate standard deviations (SD).

The widely used constitutive promoter PL has been engineered to form different inducible promoters such as PL_lacO1_, PL_tetO1_, and PL_ara_. These inducible promoters were constructed by replacing the upstream 19 bp sequence before the −35 region (Position 1) and the 17 bp sequence between the −35 region and −10 region (Position 2) with the corresponding binding sequence (e.g., LacO box and tetO box).^23^ Besides, previous studies have demonstrated that replacing the DNA sequence after the −10 region (Position 3) would also affect promoter intensity.^24^ Based on this design principle, we constructed six hybrid promoters by placing the binding box on *Pp*PcaO (15 bp) and the binding box on *Ps*PcaO (15 bp) in three locations, as shown in Figure 4a. The dose–response of six hybrid promoters (*Pp*HP1, *Pp*HP2, *Pp*HP3, *Ps*HP1, *Ps*HP2, and *Ps*HP3) was investigated upon the induction of elevated succinate and in combination with different PcaR regulators. When *Pp*HPs were cotransferred with *Pp*PcaR or *Ps*PcaR, all paired biosensor systems exhibited various levels of activation intensity and no repression without the induction of succinate (Figure 4b–d). The broadest dynamic range was obtained in the *Ps*PcaR-*Pp*HP3 group, ranging from 4288 au to 10914 au (nearly 24-fold improvement) (Figure 4d). This result indicated that both *Pp*PcaR and *Ps*PcaR function remained the same when we shifted the positions of the *Pp*PcaO binding box. When *Ps*HPs were cotransferred with *Pp*PcaR or *Ps*PcaR, the function of PcaR biosensor systems was successfully changed. Specifically, in *Pp*PcaR-*Ps*HP3 and *Ps*PcaR-*Ps*HP3 groups, the fluorescence intensity gradually decreased with the increase of succinate concentration, demonstrating that both *Pp*PcaR and *Ps*PcaR could not enhance transcription and instead served as repressors (Figure 4 e–g). Notably, the fluorescence intensity decreased from 8257 au to 1567 au in the *Ps*PcaR-*Ps*HP3 group, achieving approximately 86% transcription repression (Figure 4g). This result elucidated that the movement of RNAP was hindered when the regulator overlapped with either binding box at Position 3, causing gene repression rather than activation (Figure 4a). In addition, promoter transcription would not be repressed but somewhat further enhanced when the regulator overlapped with the binding box due to the recruitment of RNAP. To summarize, we successfully demonstrated the dual functions of both *Pp*PcaR and *Ps*PcaR by shifting the positions of the *Ps*PcaO binding box. Specifically, *Pp*PcaR acts as a repressor when paired with its native *Pp*PcaO promoter but functions as an activator when coexpressed with the *Ps*PcaO promoter or hybrid promoters with binding boxes at upstream positions (e.g., *Pp*HP1 and *Pp*HP2). Conversely, *Ps*PcaR generally serves as an activator with both *Ps*PcaO and *Pp*PcaO but shifts to a repressor mode when bound to promoters where the operator box overlaps with the −10 region or downstream (e.g., *Ps*HP3). This dual functionality is further influenced by the spatial arrangement of the binding site relative to the core promoter elements, suggesting that the transcriptional outcome is governed by the interplay between regulator–promoter pairing and the architecture of the regulatory sequence.

### 3.5 Establishment of a Bifunctional Regulation Circuit Based on PcaR

Given PcaR’s regulatory mechanism, we hypothesized that a bifunctional regulation system could be established solely using PcaR, eliminating the need for additional gene regulation systems such as antisense RNA or CRISPR. To evaluate this, we utilized RFP (red fluorescence) as the reporter for the activation module, controlled by either *Pp*HP3 or *Ps*HP1 (Figure 5a). These activation modules were then paired with repression modules regulated by *Ps*HP3, resulting in two bifunctional circuits, B1 and B2 (Figure 5a). Experimental validation demonstrated that both circuit designs successfully achieved succinate-triggered bifunctional regulation, confirming the compatibility of the activation and repression modules. Notably, each version of the circuit exhibited distinct regulatory responses (Figure 5b,c). Specifically, upon exposure to 0–1.5 g/L succinate, B1 (*Pp*HP3 + *Ps*HP3) exhibited a 4.5-fold increase in RFP expression (from 2,684 to 10,037 au), while repressing eGFP expression by 4.3-fold (from 8,634 to 2,065 au) (Figure 5b). These regulatory shifts were more pronounced than those observed in single-function circuits, likely due to *Ps*PcaR being divided between two promoters, reducing leaky binding (Figure 5b,c). A similar trend was observed in B2 (*Ps*HP1 + *Ps*HP3), where RFP activation increased 3-fold (from 3,024 to 9,054 au), and eGFP repression occurred at a 4.2-fold rate (from 7,923 to 1,945 au) in response to 0–1.5 g/L succinate (Figure 5c). Overall, these findings highlight the successful development of a versatile bifunctional regulation system using a single PcaR regulator, significantly enhancing its applicability.

**Figure 5:**
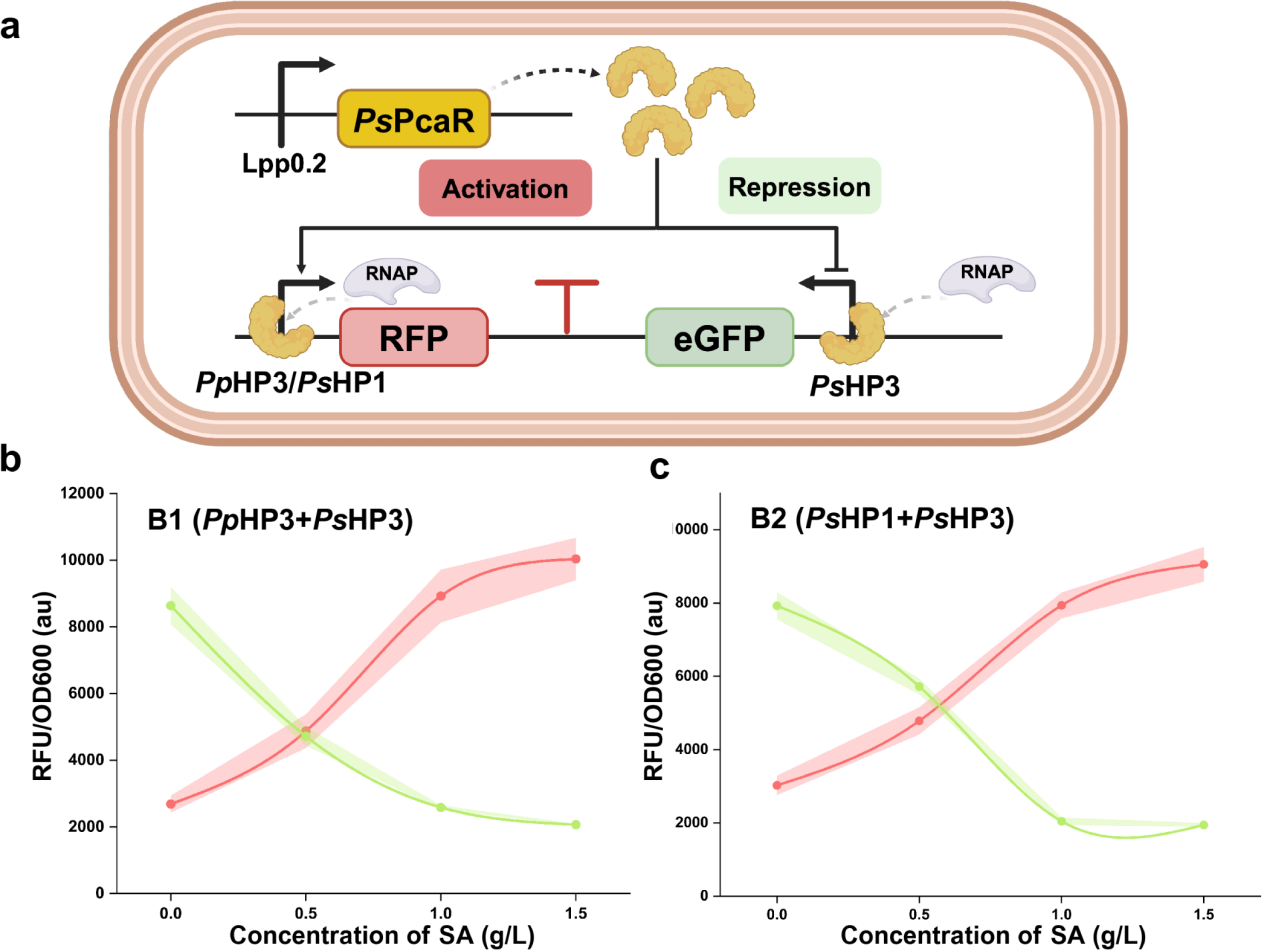
Establishment and characterization of PcaR-controlled bifunctional circuit. (a) Schematic of the bifunctional regulation system. (b) Dose–response analysis of B1 (*Pp*HP3 + *Ps*HP3). (c) Dose–response analysis of B2 (*Ps*HP1 + *Ps*HP3). All tests were performed with three independent biological repeats, and error bars indicate standard deviations (SD).

## 4 Discussion

This study characterized and engineered a succinate-responsive biosensor system based on the transcription factor PcaR, addressing the limited diversity of TF-based biosensors for central metabolic intermediates. Initial in vivo characterization revealed the restricted output strength of the wild-type *Pp*PcaR biosensor system, which limited its practical application. By fine-tuning regulator expression, we successfully restored the dynamic response of *Pp*PcaO to succinate induction. Additionally, rational engineering through site-directed mutagenesis generated the P1-AII variant, which exhibited a 32.9-fold expanded dynamic range compared to the wild-type biosensor. Promoter engineering further enabled the development of a bifunctional regulatory circuit controlled by a single regulator and succinate, demonstrating its potential in dynamic metabolic regulation. These engineered variants and regulatory circuits hold significant potential for improving pathway optimization and enhancing microbial production efficiency. However, the underlying molecular mechanism of PcaR has remained largely unexplored. Our study revealed that the dual-function mechanism of PcaR members suggests functional plasticity within the regulatory network. Through site-directed mutagenesis, we identified key residues involved in regulatory control, expanding our understanding of PcaR’s mechanism and underscoring its potential for engineering biosensors that respond to other key intermediates in central metabolism. Importantly, this study reveals that both *Pp*PcaR and *Ps*PcaR possess conditional dual functionality, with their regulatory mode determined by promoter context and binding-site location, rather than being intrinsically fixed. This expands our understanding of the functional flexibility of IclR transcription factors and offers new opportunities for tailored biosensor design.

Despite these advancements, several challenges remain. One limitation of the PcaR biosensor system is its relatively broad substrate scope. In addition to succinate, it responds to various dicarboxylic acids, which may lead to unintended cross-talk when applied in practical settings, such as dynamic metabolic regulation. This lack of specificity could hinder its broader applicability, particularly in contexts requiring precise metabolic control. In particular, the strong response to MAO highlights an interesting case of ligand preference. However, this cross-reactivity poses two key challenges: first, strong off-target activation by MAO could interfere with the sensor’s performance in dynamic regulation systems; second, MAO shows toxicity in *E. coli* at effective concentrations, impairing cell growth and limiting its applicability in practical settings. Future engineering efforts should focus on enhancing substrate specificity through rational design or directed evolution to minimize interference and improve its utility in metabolic engineering applications. Additionally, further investigations are needed to fully elucidate PcaR’s regulatory mechanism, particularly the structural basis of operator binding box recognition and its impact on PcaR conformation. The precise protein–ligand dynamics between *Ps*PcaR and succinate also require further characterization. Structural studies, such as the crystallization of *Ps*PcaR in complex with its ligand and DNA, provide valuable insights into its molecular function and inform future engineering strategies. A recent example illustrating such an approach is the in-depth structural analysis of a protein-based sensor evolved for methane activation, which thoroughly characterized the ligand-binding pocket using succinate derivatives as analogs to guide specificity design.^33^

Developing central metabolism-responsive biosensors represents a promising direction in metabolic engineering, offering real-time control over intracellular metabolic states.^34–40^ While previous studies have primarily focused on pathway-specific biosensors, our work demonstrates that targeting key intermediates, such as succinate, enables broader metabolic regulation. The engineered PcaR biosensor system provides a versatile tool for dynamic pathway regulation, high-throughput screening, and adaptive laboratory evolution. Moving forward, further expansion of central metabolism-responsive biosensors will enhance the metabolic engineering toolbox, facilitating the development of more efficient and adaptable microbial cell factories. By refining biosensor specificity and integrating advanced regulatory circuits, future studies can unlock even greater potential for dynamic control in synthetic biology applications.

## Supplementary Material



## Notes

Figure S1, Toxicity evaluation of succinic acid in *E. coli;*Figure S2, Dose–response of PcaR biosensor system to sodium succinate;Figure S3, Basal expression level of *Ps*PcaO without regulator expression; Figure S4, Dose–response of *Ps*PcaR biosensor system under lpp1.0 control;Figure S5, Time course experiments of PcaR biosensor systems by adding 0.5 g/L and 1.0 g/L succinate;Figure S6, Sequence alignment of *Pp*PcaR and *Ps*PcaR highlighting ligand-binding residues;Figure S7, Dose–response of additional *Pp*PcaR mutants;Figure S8, Dose–response of *Ps*PcaR mutants;Table S1, List of bacterial strains and plasmids used in this study;Table S2, DNA sequences of promoters and PcaR homologues used in biosensor construction;Table S3, Geometric properties of hydrogen bonds between *Pp*PcaR and succinate (PDF)
