# Supplementary material for: Characterizing and Engineering a Succinate-Responsive Biosensor System in Escherichia coli
Source: ACS Synth Biol. 2025 Aug 8;14(9):3510–9. doi: 10.1021/acssynbio.5c00290 (PMC12455658; doi:10.1021/acssynbio.5c00290)
Supplement: Supplementary file 1 [file sb5c00290_si_001.pdf]

Supplementary Information for

Characterizing and Engineering a Succinate-responsive Biosensor System

Yusong Zou[1], Yuanxin Qian[1], Connor Parish[1], Logan Huddle[1], Yajun Yan[1*]

[1] School of Chemical, Materials and Biomedical Engineering, College of Engineering, The University of Georgia, Athens, GA, 30602, USA

[*]Correspondence: yajunyan@uga.edu

Address: 2040E Interdisciplinary STEM Research Building 1, 302 East Campus Road, The University of Georgia, Athens, GA, 30602, USA

Supplementary Table 1. Strains and plasmids used in this study.

Strain	Genotype	Citation
E. coli XL-1Blue	*recA1 endA1gyrA96thi-1hsdR17supE44relA1lac [F' proAB lacIqZDM15Tn10 (TetR)]*	Stratagene
E. coli BW25113 F'	*rrnBT14 ΔlacZWJ16 hsdR514 ΔaraBADAH33 ΔrhaBADLD78 F' [traD36 proAB lacIqZΔM15 Tn10(Tetr)]*	[1]
**Plasmid**	**Description**	**Citation**
pHA-MCS	pLlacO1, colE ori, luc, Amp^r, and multiple cloning site	[2]
pHA-MCS-egfp	pHA-MCS harboring *egfp* controlled by pLlacO1	[2]
pMK-MCS	pLlacO1, p15A ori, Kan^r, and multiple cloning site	[3]
pHA-*Pp*PcaO-eGFP	pHA-MCS-eGFP under the control of *Pp*PcaO	This study
pHA- *Ps*PcaO -egfp	pHA-MCS-eGFP under the control of *Ps*PcaO	This study
pMK-Lpp1.0-*Pp*PcaR	pMK-MCS with *Pp*caR under the control of Lpp1.0	This study

pMK-Lpp0.5- *Pp*PcaR	pMK-MCS with *Pp*caR under the control of Lpp 0.5	This study
pMK-Lpp0.2- *Pp*PcaR	pMK-MCS with *Pp*caR under the control of Lpp0.2	This study
pMK-Lpp0.03- *Pp*PcaR	pMK-MCS with *Pp*caR under the control of Lpp0.03	This study
pMK-Lpp1.0- *Ps*PcaR	pMK-MCS with *Ps*caR under the control of Lpp1.0	This study
pMK-Lpp0.2- *Ps*PcaR	pMK-MCS with *Ps*caR under the control of Lpp0.2	This study
pMK-Lpp0.2- *Pp*PcaR (Y151A)	*Pp*PcaR was replaced by its mutant Y151A in pMK-Lpp0.2- *Pp*PcaR	This study
pMK-Lpp0.2- *Pp*PcaR (N257I/S259I)	*Pp*PcaR was replaced by its mutant N257I/S259I in pMK-Lpp0.2- *Pp*PcaR	This study
pMK-Lpp0.2- *Pp*PcaR (Y151A/N257I/S259I)	*Pp*PcaR was replaced by its mutant Y151A/N257I/S259I in pMK-Lpp0.2- *Pp*PcaR	This study
pHA-*Pp*HP1-eGFP	pL_{lacO} was replaced hybrid promoter *Pp*HP1 in pHA-MCS-eGFP	This study
pHA-*Pp*HP2-eGFP	pL_{lacO} was replaced hybrid promoter *Pp*HP2 in pHA-MCS-eGFP	This study
pHA-*Pp*HP3-eGFP	pL_{lacO} was replaced hybrid promoter *Pp*HP3 in pHA-MCS-eGFP	This study

pHA-*Ps*HP1-eGFP	pL_{lacO} was replaced hybrid promoter *Ps*HP1 in pHA-MCS-eGFP	This study
pHA-*Ps*HP2-eGFP	pL_{lacO} was replaced hybrid promoter *Ps*HP2 in pHA-MCS-eGFP	This study
pHA-*Ps*HP3-eGFP	pL_{lacO} was replaced hybrid promoter *Ps*HP3 in pHA-MCS-eGFP	This study
pHA-*Ps*HP3-eGFP-*Pp*HP3-RFP	pHA-*Ps*HP3-eGFP harboring with RFP under the control of *Pp*HP3	This study
pHA-*Ps*HP3-eGFP-*Ps*HP1-RFP	pHA-*Ps*HP3-eGFP harboring with RFP under the control of *Ps*HP1	This study

Supplementary Table 2. PcaR homologs sequence used in this study.

Strain	Sequence	Citation
*Pp*PdhR	atgAGTGACGAAACCCTGGGCAACGATTCCGGCAATGCTGAG<br>GTGGCGCGACCTGCTTCGGCTGCCATGGCGCCGCCGATCGTG<br>GCTTCCCCGGCCAAGCGCATCCAGGCCTTTACCGGTGACCCG<br>GACTTCATGACCTCACTGGCACGTGGCCTGGCCGTGATCCAG<br>GCATTCCAGGAGCGCAAGCGCCACCTGACCATCGCCCAGAT<br>CAGTCACCGTACCGAAATCCCTCGCGCGGCGGTGCGCCGTTG<br>CCTGCACACGTTGATCAAACTGGGTTACGCCACCACCGACGG<br>GCGTACCTATTCGCTGCTGCCCAAAGTGCTGACCTTGGGGCA<br>TGCCTACCTGTCGTCGACGCCGCTGGCAATTTCTGCACAGCC<br>GTACCTGGATCGCATCAGCGACCAGCTGCATGAGGCGGCCA<br>ACATGGCCACCCTCGAGGGCGACGACATCCTTTATATAGCCC<br>GTTCGGCCACGGTCGAGCGGCTGATCTCGGTCGATCTGTCGG<br>TGGGCGGGCGCCTGCCGGCCTATTGCACGTCGATGGGGCGC<br>ATTCTGCTGGCGGCCATGGATGACACCAGCCTGCGTGAGTAC<br>CTGGAACGTGCCGACCTCAAAGCGCGCACCAGCCGCACCCT<br>GAATGACCCCGAATCGTTGTTCGCCTGCATCCAGCAGGTGCG<br>CGCACAGGGCTGGTGTGTGGTAGACCAGGAACTGGAACAGG<br>GGCTGCGATCGATTGCCGTGCCGGTGTATGACGCTTCGGGGC<br>AGGTGTTGGCTGCGCTGAACGTCAGTACCCATGTCGGGCGG<br>GTGACCCGCAGTGAACTGGAGCAGCGGTTCTTGCCGATTTTG<br>CTGGCAGCCAGCCGGGACCTTTGCCACCAGTTGTTTGGTtga	This study

*Pp*PcaO	ATGGGAAAGGTATAAGCAAAAGTGTCGGCGGTCAATTGCGA TTATCGGCCGTTTGTTCGATAATCGCACGAAC	This study
*Pp*PcaO binding box	GTTCGATAATCGCAC	This study
*Ps*PcaR	atgGCAGACAGCAACGCGGACACGGCGCCACGGCGGCAGAAG GTACAGGCCGCAGAGGTAGGTACTGACATTCTCACTGCCCTG GCCGAGCTGGCCCCGGCGACCTCCTTATCGCGCCTGGCCGAA CATGTCGGCATGCCTGCCAGCAAGGTCCATCGCTATTTGCAG GCCCTGATGGCGAGCGGCTTCGCCGAGCAGGATCCCCTGAC GAACCATTACGGGCTGGGCCGCGCCGCGCTGTTCGTGGGCTT GGCCGCGTTGGGCCGACTGGATGTCGTCAAACTGGCAACGC CGCATCTGGCGCAGCTGCGTGATGAACTCAACGAAACCTGCT TTCTTGCGGTTTGGGGTAACCGCGGTCCGGCCGTCGTGCATG TCGAACAGGCGGTCCGTGCCGTGACGCTGGTGACACAGGTG GGCTCGGTGCTGCCTTTGCTGGGCTCATCGACCGGGTTGGTG TTCAACGCCTTCATGCCCAACGCCGAGACTGCGCAATTGCGC GAAGAGGAACTGAAGCTTCCCTCGGCACCCAGCCCTGCAGC CCTGCTCGCTGCCATGAGCGAACTGCAACGCACCCACATCCA GCCCGTGCACGGCCTGTTGATGGCTGGCGTCAACGCACTCTC CGCGCCGCTGTTCAGTGGCGACCAGCGGTTGGCAGGCGTGA TCACCATCGTCGGTGGCGAGCCGGGCTTCATGGCCGAAGCG	This study

	GATGGCGAGGCGGCAAAGCGACTGCTGACAGTGGCGCGCAA GATCAGCGCGCGGATGGGTGCCtaa	
*Ps*PcaO	TGCGTTTACGCAAAAAGTAATTTGAACTCACCAGGGCGTCAA GTTATAAACAGCGCCTCGATAAGCGAGCC	This study
*Ps*PcaO binding box	GCCTCGATAAGCGAG	This study

Supplementary Table 3. Geometric properties of hydrogen bonds between *Pp*PcaR and succinate (PDB ID: 8eju).

Residue (PpPcaR)	Interacting Atom (Residue)	Interacting Atom (Succinate)	Donor–Acceptor Distance (Å)	Bond Angle (°)
Y151	OH	O1	2.9	158
T177	OG1	O2	3.0	162
S240	OG	O1	3.2	145
N257	ND2	O2	2.8	160
S259	OG	O1	3.1	148

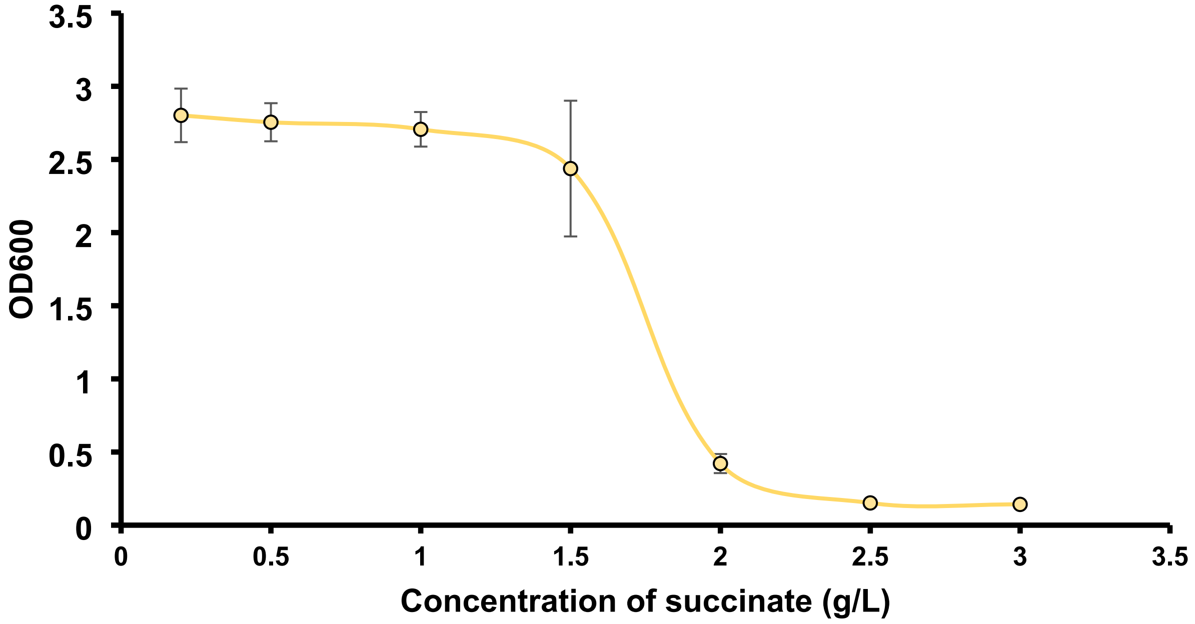

Supplementary Figure S1. SA toxicity. All error bars represent standard deviation (n = 3). The experiments are biological replicates.

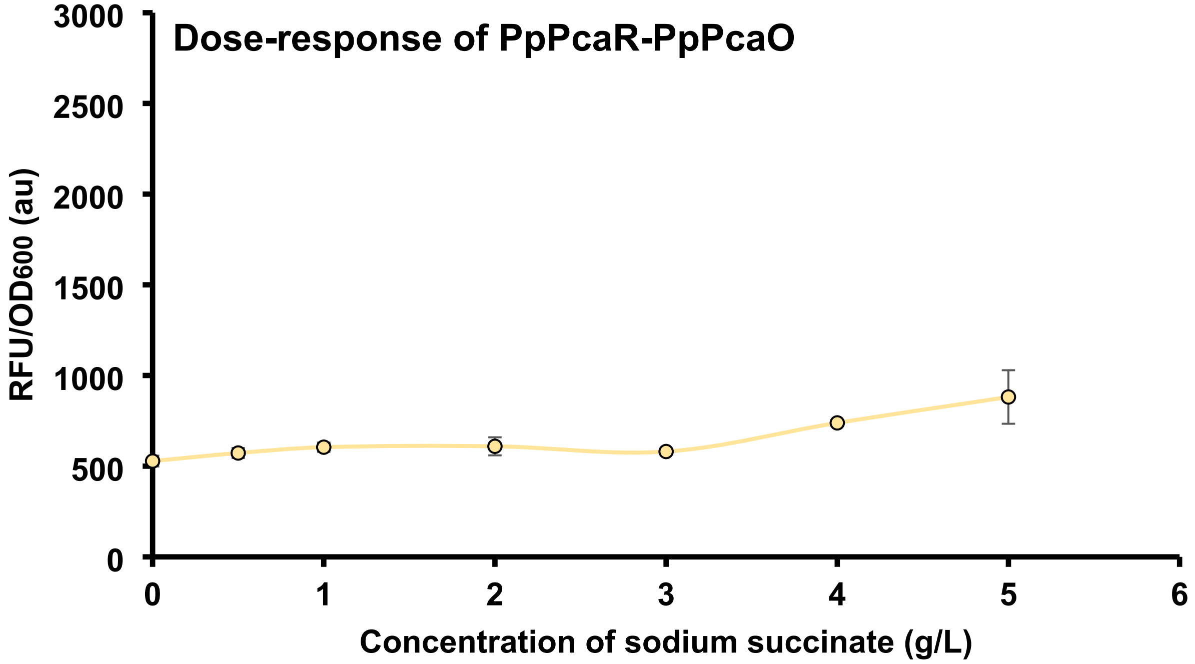

Supplementary Figure S2. Dose-response of *Pp*PcaR-*Pp*PcaO upon the induction of sodium succinate. All error bars represent standard deviation (n = 3). The experiments are biological replicates.

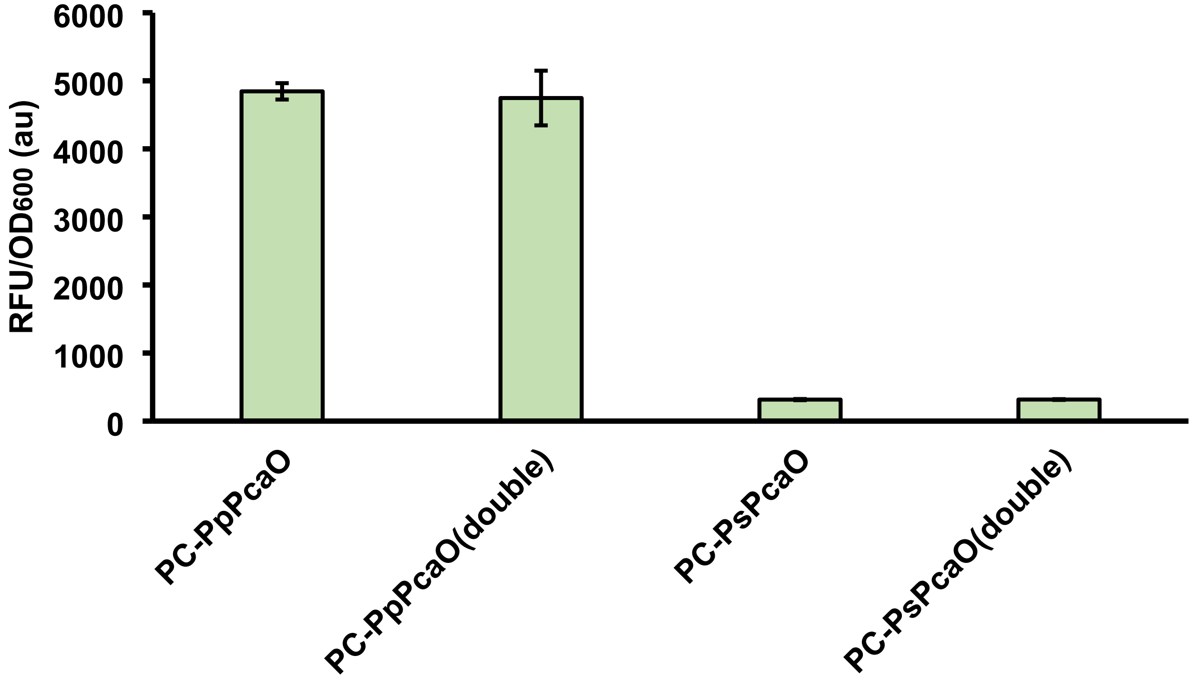

Supplementary Figure S3. Intensity of original PcaO promoters. PC-PpPcaO, the original intensity of *Pp*PcaO promoter. PC-PpPcaO (double), the original intensity of *Pp*PcaO promoter harboring with pMK-MCS. PC-PsPcaO, the original intensity of *Ps*PcaO promoter. PC-PsPcaO (double), the original intensity of *Ps*PcaO promoter harboring with pMK-MCS. All error bars represent standard deviation (n = 3). The experiments are biological replicates.

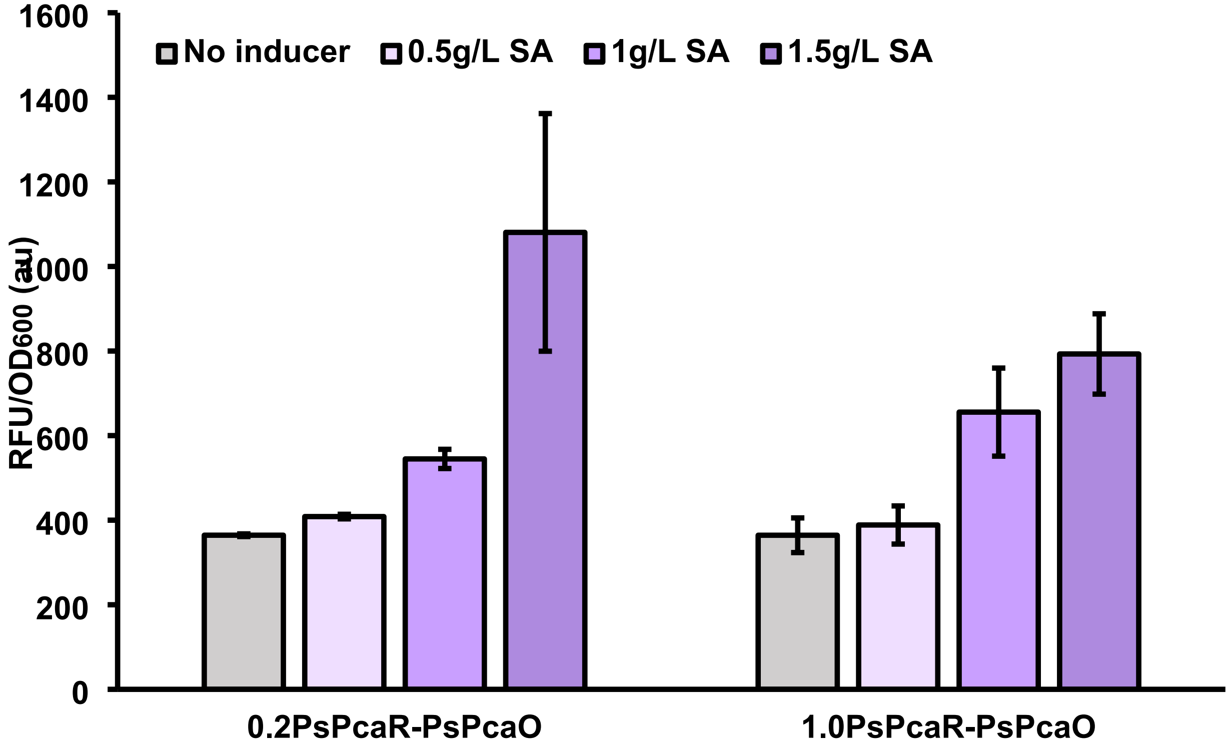

Supplementary Figure S4. Dose-response of *Ps*PcaR biosensor systems. All error bars represent standard deviation (n = 3). The experiments are biological replicates.

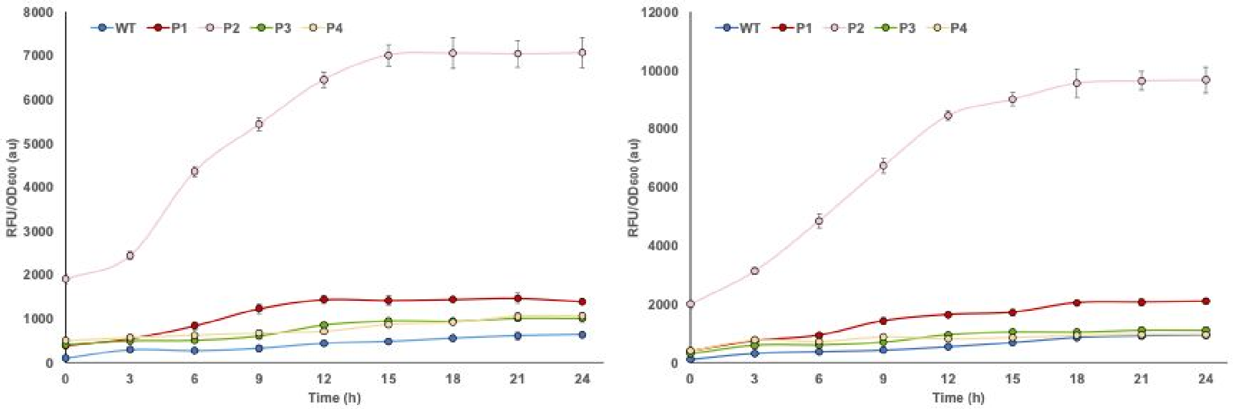

Supplementary Figure S5. Time course experiment of paired PcaR biosensor systems under the induction of 0.5 g/L and 1.0 g/L succinate. All the tests were performed with three independent biological repeats, and error bars indicate standard deviations (SD).

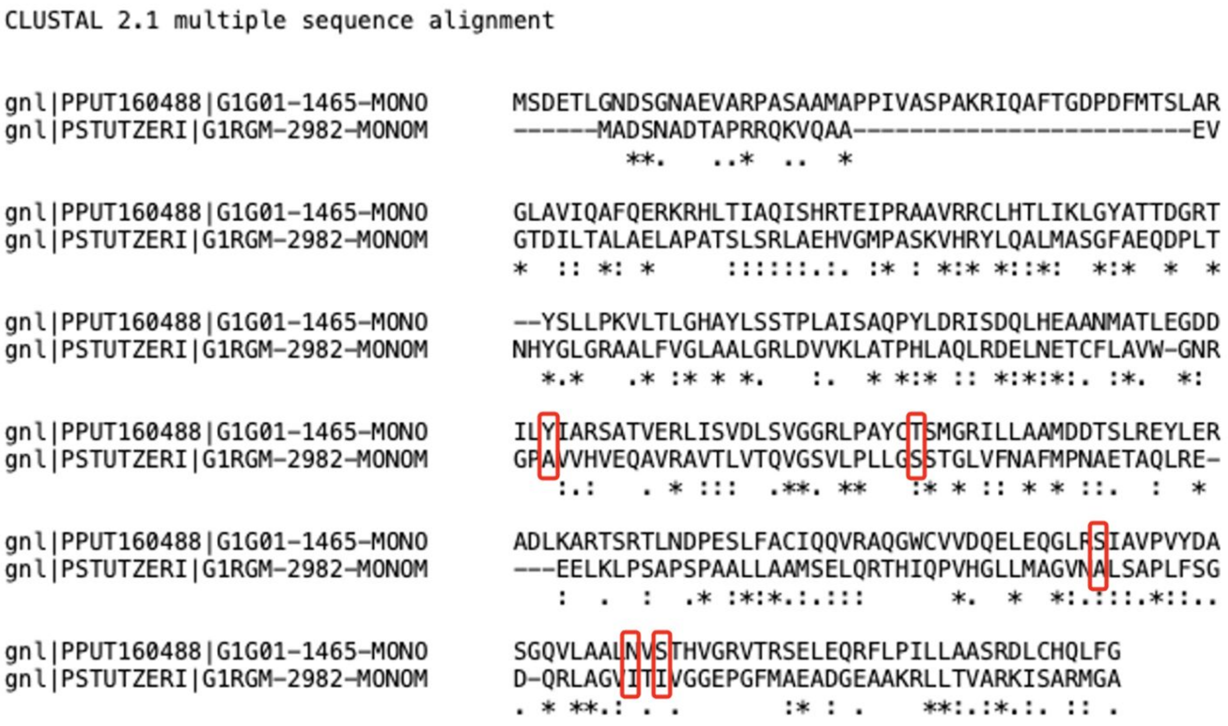

```
CLUSTAL 2.1 multiple sequence alignment

gnl|PPUT160488|G1G01-1465-MONO      MSDETLGNDSGNAEVARPASAAMAPPIVASPAKRIQAFTGDPDFMTSLAR
gnl|PSTUTZERI|G1RGM-2982-MONOM      ------MADSNADTAPRRQKVQAA------------------------EV
                                             **.    ..*   ..  *

gnl|PPUT160488|G1G01-1465-MONO      GLAVIQAFQERKRHLTIAQISHRTEIPRAAVRRCLHTLIKLGYATTDGRT
gnl|PSTUTZERI|G1RGM-2982-MONOM      GTDILTALAELAPATSLSRLAEHVGMPASKVHRYLQALMASGFAEQDPLT
                                    *  :: *: *      ::::::.:. :* : *:* *::*:   *:*  *  *

gnl|PPUT160488|G1G01-1465-MONO      --YSLLPKVLTLGHAYLSSTPLAISAQPYLDRISDQLHEAANMATLEGDD
gnl|PSTUTZERI|G1RGM-2982-MONOM      NHYGLGRAALFVGLAALGRLDVVKLATPHLAQLRDELNETCFLAVW-GNR
                                      *.*   .* :* * *.    :.   * *:* :: *:*:*:. :*.   *:

gnl|PPUT160488|G1G01-1465-MONO      ILYIARSATVERLISVDLSVGGRLPAYCTSMGRILLAAMDDTSLREYLER
gnl|PSTUTZERI|G1RGM-2982-MONOM      GPAVVHVEQAVRAVTLVTQVGSVLPLLGSSTGLVFNAFMPNAETAQLRE-
                                       :.:    . * :::  .**. **    :* * :: * * ::.   :  *

gnl|PPUT160488|G1G01-1465-MONO      ADLKARTSRTLNDPESLFACIQQVRAQGWCVVDQELEQGLRSIAVPVYDA
gnl|PSTUTZERI|G1RGM-2982-MONOM      ---EELKLPSAPSPAALLAAMSELQRTHIQPVHGLLMAGVNALSAPLFSG
                                       :   .   :   .* :*:*.:.:::       *.   *   *:.:::.*::..

gnl|PPUT160488|G1G01-1465-MONO      SGQVLAALNVSTHVGRVTRSELEQRFLPILLAASRDLCHQLFG
gnl|PSTUTZERI|G1RGM-2982-MONOM      D-QRLAGVITIVGGEPGFMAEADGEAAKRLLTVARKISARMGA
                                    . * **.: . .         :* : .     **:.:*.:. :: .
```

Supplementary Figure S6. Alignment of *Pp*PcaR and *Ps*PcaR. *Pp*PcaR, Protein sequence of G1G01-1465; *Ps*PcaR, Protein sequence of G1RGM-2982. Aligned critical residues were highlighted with red circle.

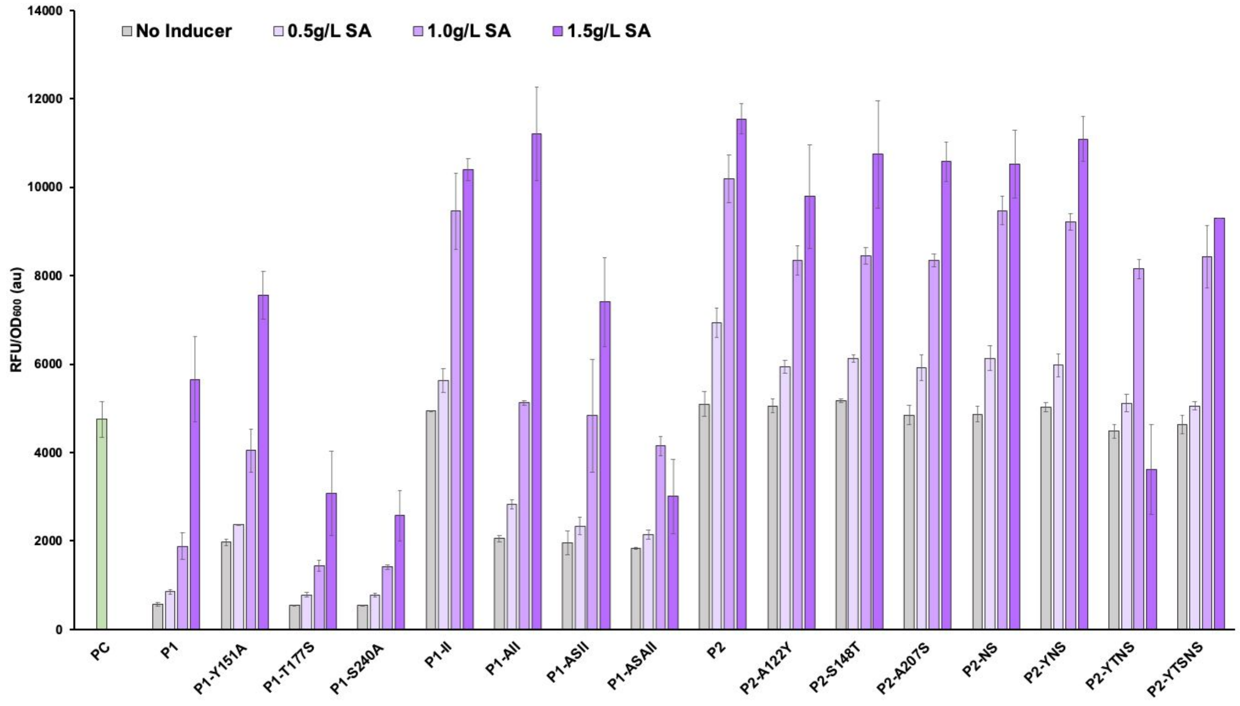

Supplementary Figure S7. Dose-response of site-directed mutagenesis on *Pp*PcaR and *Ps*PcaR harboring with *Pp*PcaO. PC, intensity of *Pp*PcaO. P1, lpp0.2 controlled *Pp*PcaR-*Pp*PcaO. P1-Y151A, lpp0.2 controlled *Pp*PcaR (Y151A)-*Pp*PcaO. P1-T177S, lpp0.2 controlled *Pp*PcaR (T177S)-*Pp*PcaO. P1-S240A, lpp0.2 controlled *Pp*PcaR (S240A)-*Pp*PcaO. P1-II, lpp0.2 controlled *Pp*PcaR (N257I/S259I)-*Pp*PcaO. P1-AII, lpp0.2 controlled *Pp*PcaR (Y151A/N257I/S259I)-*Pp*PcaO. P1-ASII, lpp0.2 controlled *Pp*PcaR (Y151A/T177S/N257I/S259I)-*Pp*PcaO. P1-ASAII, lpp0.2 controlled *Pp*PcaR (Y151A/T177S/S240A/N257I/S259I)-*Pp*PcaO. P2, lpp0.2 controlled *Ps*PcaR-*Pp*PcaO. P2-A122Y, lpp0.2 controlled *Ps*PcaR (A122Y)-*Pp*PcaO. P2-S148T, lpp0.2 controlled *Ps*PcaR (S148T)-*Pp*PcaO. P2-A207S, lpp0.2 controlled *Ps*PcaR (A207S)-*Pp*PcaO. P2-NS, lpp0.2 controlled *Ps*PcaR (I223N/I225S)-*Pp*PcaO. P2-YNS, lpp0.2 controlled *Ps*PcaR (A122Y/I223N/I225S)-*Pp*PcaO. P1-ASII, lpp0.2 controlled *Pp*PcaR (A122Y/S148T/I223N/I225S)-*Pp*PcaO. P2-ASAII, lpp0.2 controlled *Ps*PcaR

(A122Y/S148T/A207S/I223N/I225S)-*Pp*PcaO. All error bars represent standard deviation (n = 3). The experiments are biological replicates.

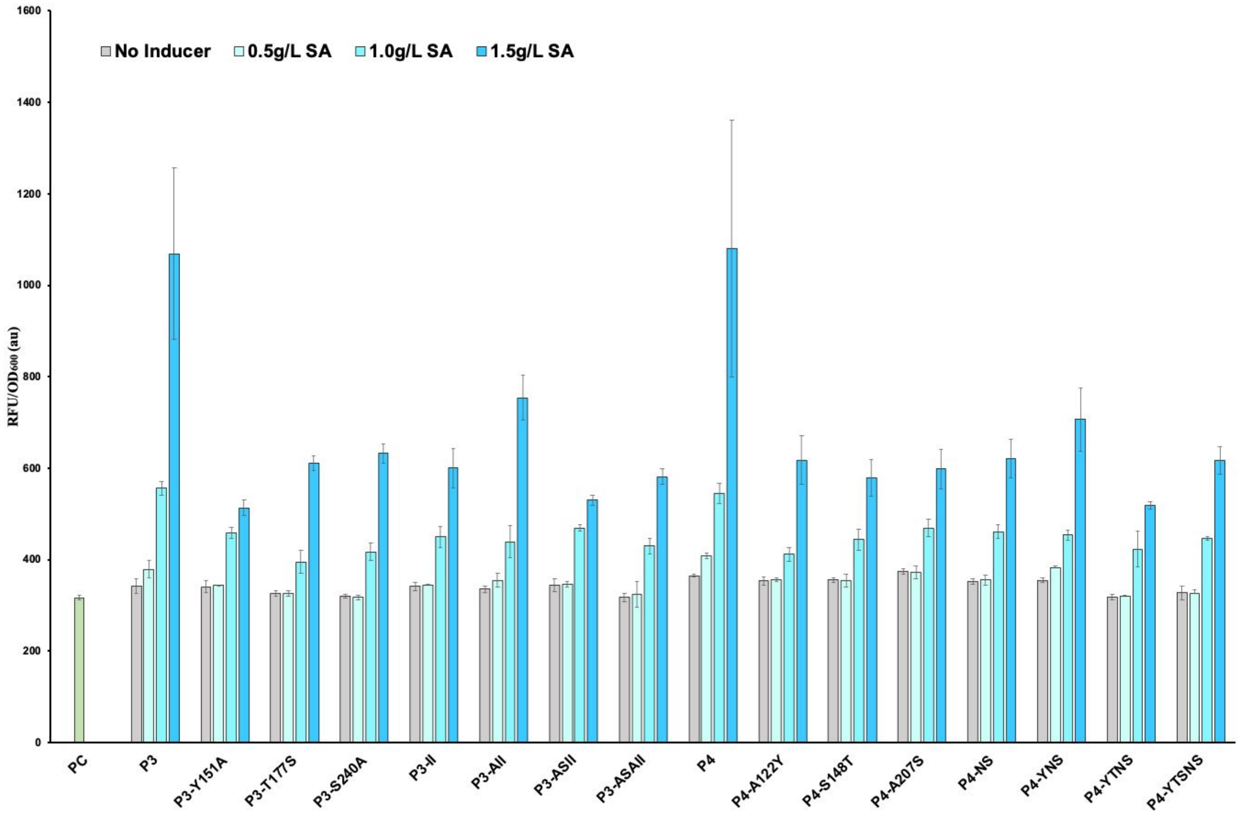

Supplementary Figure S8. Dose-response of site-directed mutagenesis on *Pp*PcaR and *Ps*PcaR harboring with *Ps*PcaO. PC, intensity of *Ps*PcaO. P3, lpp0.2 controlled *Pp*PcaR-*Ps*PcaO. P3-Y151A, lpp0.2 controlled *Pp*PcaR (Y151A)-*Ps*PcaO. P3-T177S, lpp0.2 controlled *Pp*PcaR (T177S)-*Ps*PcaO. P3-S240A, lpp0.2 controlled *Pp*PcaR (S240A)-*Ps*PcaO. P3-II, lpp0.2 controlled *Pp*PcaR (N257I/S259I)-*Ps*PcaO. P3-AII, lpp0.2 controlled *Pp*PcaR (Y151A/N257I/S259I)-*Ps*PcaO. P3-ASII, lpp0.2 controlled *Pp*PcaR (Y151A/T177S/N257I/S259I)-*Ps*PcaO. P3-ASAII, lpp0.2 controlled *Pp*PcaR (Y151A/T177S/S240A/N257I/S259I)-*Ps*PcaO. P4, lpp0.2 controlled *Ps*PcaR-*Ps*PcaO. P4-A122Y, lpp0.2 controlled *Ps*PcaR (A122Y)-*Ps*PcaO. P4-S148T, lpp0.2 controlled *Ps*PcaR (S148T)-*Ps*PcaO. P4-A207S, lpp0.2 controlled *Ps*PcaR (A207S)-*Ps*PcaO. P4-NS, lpp0.2 controlled *Ps*PcaR (I223N/I225S)-*Ps*PcaO. P4-YNS, lpp0.2 controlled *Ps*PcaR

(A122Y/I223N/I225S)-*Ps*PcaO. P4-ASII, lpp0.2 controlled *Pp*PcaR (A122Y/S148T/I223N/I225S)-*Ps*PcaO. P4-ASAII, lpp0.2 controlled *Ps*PcaR (A122Y/S148T/A207S/I223N/I225S)-*Ps*PcaO. All error bars represent standard deviation (n = 3). The experiments are biological replicates.

References

1. Atsumi, S., Anthony F. C., Michael R. C., Claire R. S., Kevin M. S., Mark P. B., Katherine JY C., Taizo H., James C. L. (2008) Metabolic engineering of Escherichia coli for 1-butanol production. *Metab Eng. 10* (6), 305-311.
2. Jiang, T., Li, C., Zou, Y., Zhang, J., Gan, Q., Yan, Y. (2022) Establishing an Autonomous Cascaded Artificial Dynamic (Auto-CAD) regulation system for improved pathway performance. *Metab. Eng. 74*, 1–10.
3. Zou, Y., Zhang, J., Wang, J., Gong, X., Jiang, T., Yan, Y. (2024) A self-regulated network for dynamically balancing multiple precursors in complex biosynthetic pathways. *Metab. Eng. 82*, 69–78.
